# Quantitative organization of the excitatory synapses of the primate cerebellar nuclei: further evidence for a specialized architecture underlying the primate cerebellum

**DOI:** 10.1007/s00429-019-01888-8

**Published:** 2019-05-17

**Authors:** Haian Mao, Salah Hamodeh, Angelos Skodras, Fahad Sultan

**Affiliations:** 1 Department of Cognitive Neurology, Hertie Institute for Clinical Brain Research, Otfried-Müller-Str. 27, 72076 Tübingen, Germany; 2 Department of Cellular Neurology, Hertie Institute for Clinical Brain Research, Otfried-Müller-Str. 27, 72076 Tübingen, Germany; 3 Department of Integrative Medical Biology, Umeå University, Linnéus Väg 9, 901 87 Umeå, Sweden

**Keywords:** Vesicular glutamate transporter, Lipofuscin fluorescence removal, Deep cerebellar nuclei, Quantitative immunofluorescence, Comparative neuroanatomy

## Abstract

The cerebellar intrinsic connectivity is of remarkable regularity with a similar build repeated many times over. However, several modifications of this basic circuitry occur that can provide important clues to evolutionary adaptations. We have observed differences in the wiring of the cerebellar output structures (the deep cerebellar nuclei, DCN) with higher dendritic length density in the phylogenetically newer DCN. In rats, we showed that an increase in wiring is associated with an increase in the presynaptic vesicular glutamate transporter 1 (vGluT1). In this study, we have extended our analysis to the rhesus monkey and can show similarities and differences between the two species. The similarities confirm a higher density in vGluT1+ boutons in the lateral (LN/dentate) and posterior interpositus nucleus compared to the phylogenetically older DCN. In general, we also observe a lower density of vGluT1 and 2+ boutons in the monkey, which however, yields a similar number of excitatory boutons per neuron in both species. The only exception is the vGlut1+ boutons in the macaque LN/dentate, which showed a significantly lower number of vGluT1+ boutons per neuron. We also detected a higher percentage of co-labelled vGluT1 and 2 boutons in the macaque than we found in the rat. In summary, these results confirm that the hyposcalled dendrites of the monkey LN/dentate also show a lower number of vGluT1+ boutons per neuron. These results provide further support of our model relating the dendritic morphology of the LN/dentate neurons to the morphology of the specially enlarged LN/dentate nucleus in primates.

**Electronic supplementary material:**

The online version of this article (10.1007/s00429-019-01888-8) contains supplementary material, which is available to authorized users.

## Introduction

The deep cerebellar nuclei (DCN) are a key component of the cerebellar circuitry and the main output of the cerebellum. The activity of DCN neurons is controlled by the integration of inhibitory synaptic input from the cerebellar cortex (i.e., the Purkinje cell axons) and excitatory input from the precerebellar nuclei. The precerebellar afferents are from a wide source of brainstem nuclei conveying vestibular, somatosensory, visual, auditory stimuli and from the basilar pontine nuclei conveying cerebral information. There are two large groups of output neurons within the DCN. An excitatory projection group that provides glutamatergic inputs to different brain regions within the brainstem (e.g., the red nucleus) and the cerebral cortex (via specific thalamic relay nuclei). In contrast, the other group is GABAergic and only targets the inferior olive nuclei. Both paths are also involved in recurrent circuits that feed back to the DCN and cerebellar cortex either via the collaterals of the mossy fibers or of the climbing fibers. In classical conditioning learning tasks, such as the Pavlovian eyeblink conditioning, the mossy fibers have been shown to convey the conditioned stimulus, whereas the climbing fibers provide the unconditioned stimulus to the DCN and cerebellar cortex (Boele et al. 2013; Lee et al. 2015). This dichotomy is further underlined by the presence of two vesicular glutamate transporters (vGluT1 and 2) used by the two paths in different ways; the climbing fibers exclusively use vGluT2, while all mossy fiber express vGluT1. It is noteworthy that a substantial subpopulation of mossy fibers also co-expresses vGluT2 (Hisano et al. 2002; Hioki et al. 2003; Boulland et al. 2004) and it is still unclear what functional role this co-labelling provides.

Based on the expression of vGluT1 and 2 in the climbing and mossy fiber DCN collaterals, immunolabeling these two transporters is an ideal way of marking all the excitatory axon terminals in the DCN. Using this approach together with systematic unbiased sampling of the rat DCN we were able to show that both transporters are expressed in the DCN in similar proportions. However, we also found that vGluT1+ bouton density is significantly higher in the phylogenetically newer lateral nucleus/dentate (LN/dentate) and posterior interpositus nucleus (PIN) compared to the phylogenetically older DCN (medial nucleus: MN and anterior interpositus nucleus: AIN). In contrast the vGluT2+ bouton density did not show any significant difference between the different DCN (Mao et al. 2018). These findings is in agreement with the general brain pattern of vGluT1 and 2 expression, with the former predominantly expressed in the phylogenetically newer brain regions (cerebral cortex) and the later in subcortical regions such as the midbrain and thalamic nuclei (Fremeau et al. 2004). Based on this view our first prediction would be that in primates with an enlarged neocerebellum the density of vGluT1+ boutons in the LN/dentate should also be increased within this nucleus. However, it is not clear whether the results from the rat LN/dentate can be generalized to primates. In fact a different prediction emerges if we look at the wiring components within the DCN. In a previous study (Hamodeh et al. 2017), we quantified and compared the wiring components between the rat and rhesus monkey DCN. We found that although the dendritic length density (dendritic length per tissue volume) was increased within the LN/dentate, a different picture emerged when we compared the dendritic length per neuron (dendritic length density normalized by neuron density). Here we found that the LN/dentate had less dendritic length per neuron than would have been predicted by the overall volume of the primate LN/dentate. We termed this observation hyposcaling of dendrites and related it to the flattened shape of the LN/dentate. Therefore, the quantification of the vGluTs in the primate LN/dentate could clarify whether we also observe a hyposcaling of the excitatory synapses in this nucleus.

Our immunofluorescence approach that we established in the rodent should also be applicable to the primate brain tissue on the basis of antigen homology (Garcia-Marin et al. 2013). However, the presence of lipofuscin complicates this approach (Terman and Brunk 2004), due to its broad emission spectrum. In this study, we, therefore, modified an approach from flow cytometry (Steinkamp and Stewart 1986; Roederer and Murphy 1986; Billinton and Knight 2001) to remove the lipofuscin emission. This required acquiring an additional lipofuscin specific channel and using that to mask the vGluT1 and vGluT2 signals during postprocessing.

## Experimental procedure

### Tissue preparation

All animal experiments were carried out in accordance with the Society for Neuroscience and local German authorities (approved by the regional authorities (Regierungspräsidium Tübingen). Brain tissue from two male adult macaques was used in this study (D98: body weight 17 kg and 18 years old, H01: body weight 9 kg and 13 years old). Following premedication with a mixture of ketamine (20 mg/100 g body weight), xylazine (2 mg/100 g body weight) deep anesthesia was induced with pentobarbital (400 mg/kg). The animals were then perfused transcardially by 0.1 M PB and then by 4% ice-cold paraformaldehyde in 0.1 M phosphate buffer (PB) at pH 7.4. The brain was immediately dissected out of the skull and then cryoprotected in an ascending concentration of sucrose (10%, 20%, and 30% in 0.1 M PB). The cerebellum was removed from the brainstem and mounted on a microtome with the lateral side facing the freezing platform. Sections of 40 µm (D98) or 50 µm (H01) were serially acquired and stored in 0.1 M PB. Immunofluorescence staining was carried out on free floating sections. Prior to primary antibody incubation, sections were washed three times in 0.1 M PB for 5 min each time and then blocked in 0.1 M PB with 10% horse serum (PAA Laboratories, Coelbe, Germany) and 0.3% Triton X-100 at room temperature for 1 h.

### Immunofluorescence

The concentration and incubation times were tested for each antibody in single stains. The optimized conditions were then used for the multiple stains. We performed a quadruple staining protocol for vGluT1, vGluT2, Purkinje Cell Protein 2(PCP2), and potassium channel KCNC3/Kv3.3 on H01, and a further quadruple staining protocol for vGluT1, vGluT2, MAP2 and KCNC3/kv3.3 on D98. Data of the Kv3.3 were used for a subsequent publication. The sections were first incubated with goat vGluT1 (sc-13320, Santa Cruz Biotechnology, Texas) at 1:1000 and 4 °C for 16 h, followed by 3 × washes in 0.1 M PB and then incubated with donkey anti goat Alexa Fluor 488 at 1:500 for 2 h at room temperature. The sections were then washed three times in PB before incubation with a combination of three antibodies of vGluT2 (1:1000, lot number: 135404, Synaptic systems, North Saanich British Columbia), Kv3.3 (1:500, lot number: APC-102, Alomone labs, Israel) and PCP2 (1:200, lot number: sc-137064, Santa Cruz Biotechnology, Texas) or MAP2 (1:200, lot number: M1406, Sigma Aldrich) at 4 °C for 16 h. The sections were then washed and incubated with the combinations of the three secondary antibodies: goat anti-guinea pig Alexa Fluor 633 (1:500, A21105, Invitrogen, California), goat anti-rabbit Cy3 (1:500, lot number: 81-6115, Invitrogen, California) and goat anti-mouse Alexa Fluor 405 (1:500, lot number: A31553, Invitrogen, California) for 2 h at room temperature. Sections were then washed and mounted in glycerol on glass slides with Mowiol 4–88 (Merck, Darmstadt, Germany). The slides were stored at 4 °C.

### Preabsorption test of vGluT2 antibody

To test for vGluT2 antigen–antibody specificity, we mixed 1 μl vGluT2 antibody with 2 μl of the peptide that was used for immunization and was provided by the supplier. This consisted of Strep-Tag fusion protein (amino acids 510–582 of the rat vGluT2) in 500 μl 0.5 M PB solution with 2% horse serum and 0.1% tritonX-100. The mixture was rotated at room temperature for 1 h to allow for full interaction of the peptide with the antibody. As a control solution we used 1 μl vGluT2 antibody diluted in 500 μl 0.5 M PB solution with 2% horse serum and 0.1% tritonX-100. Two adjacent cerebellar sections from the same series of monkey H01 were washed and blocked. One section was incubated in the antigen–antibody mixture and the other in the control solution for 48 h at 4° with constant rotation. The two sections were washed and incubated with goat anti-guinea pig Alexa Flour 633 1:1000 for 2 h at room temperature. The sections were washed, mounted, and scanned with laser confocal microscope LSM 510. VGluT2 channel was scanned under the excitation of 633 nm and emission at 650–750 nm, while lipofuscin channel was scanned under the excitation of 405 nm and emission at 650–750 nm.

### Data acquisition

Images were acquired on a laser scanning confocal microscope (LSM 510, Carl Zeiss, Jena, Germany). We took overview images of vGluT1 staining for every section with 488 nm excitation and emission band pass filtering 505–550 nm at low magnification (10 × objective). Probe positions were determined by first marking an identifiable origin (*x*, *y* coordinates: 0, 0) and then determining the position of the other probes in the DCN region at a regular spacing of 1000 μm × 1000 μm. The location of the origin point was chosen from an easily identifiable structure (i.e., vessels) within a core region of the DCN. However, this location differs from slice to slice and is not related to the structures to be analyzed. A *z*-stack was acquired using a 63 × (NA1.4, Oil immersion) objective, applying a 2 × zoom for each probe. The pinhole was set to one airy unit and subsequently optimized for every detection channel to achieve an equal optical slice thickness on all channels. *XY* voxel size was set to 0.14 μm × 0.14 μm; image digital resolution was set to 512 × 512 pixels. The *z*-stack step size was 0.32 μm and we took an average of 30 optical sections. We stained and analyzed 13 cerebellar sections (5 slices from D98 and 8 slices from H01) and 168 probes in total were sampled (90 from D98, 78 from H01). The fluorescence emitted by lipofuscin granules were recorded with excitation wavelength at 405 nm and emission wavelength at 650–750 nm. Details of antibodies, excitation wavelength and emission filters are listed in Table 1.

**Table 1:** Multichannel laser confocal microscopy parameters

1st antibody	2nd antibody	Excitation (nm)	Emission (nm)	Track number
Goat vGluT1	Donkey anti-goat Alexa flour 488	488	505–550	1
Guinea pig vGluT2	Goat anti-guinea pig Alexa flour 633	633	650–750	1
Mouse PCP2 or mouse MAP2	Goat anti-mouse Alexa flour 405	405	420–480	2
Rabbit kv3.3	Goat anti rabbit cy3	543	560–615	2
None	None	405	650–750	3

Four different secondary antibodies were used with the corresponding primary antibodies. The fluorophores were excited at their optimal wavelength and the emissions were collected using different band pass or long pass filters. Lipofuscin was excited such as to obtain maximal difference between excitation and emission wave length, thereby dissociating it from the other fluorophores. Three tracks were used during the acquisition process to minimize channel interference

We also determined tissue shrinkage in section thickness by taking four random positions within DCN region for each slice and determining the upper and lower section borders by the staining signal for vGluT1 at a magnification of 20x (NA0.8, dry). Fifty-two probes were taken from 13 sections. The mean thickness of the slices after mounting was 24.6 μm for D98 and 26 μm for H01, while the original sectioning thickness was 40 μm and 50 μm, respectively. Classification of the macaca DCN was based on the description in an earlier paper (Hamodeh et al. 2014).

### Data preprocessing and analysis

Data were acquired as 8-bit format and saved in Zeiss lsm format. Stacks were then deconvoluted using the iterative (*n* = 10) “blind deconvolution” of AutoQuant X3 (Media Cybernetics, Bethesda, MD) with the maximum likelihood estimation and constrained iteration. Following deconvolution, data were saved in Autoquant to 8-bit lsm format.

### Lipofuscin masking and extraction of vGluTs boutons

Lipofuscin has a broader excitation and emission spectrum than commercial fluorophores which are designed with narrow spectra. We acquired the signals from our own fluorophores by regular microscopic settings while simultaneously recording lipofuscin fluorescence signal in an additional channel and subtracted the lipofuscin fluorescence during post-processing.

We performed surface reconstruction to get boundaries of lipofuscin granules using the Imaris software (Bitplane). The lipofuscin surfaces were smoothed at 0.279 μm and the background was subtracted using the Ridler and Calvard (R–C) algorithm (Ridler and Calvard 1978) to determine the best thresholding level for this channel. The thresholding level was about 35/255. The reconstructed lipofuscin surfaces were subsequently used to mask the vGluT1 and vGluT2 channel. The voxels inside the lipofuscin mask were set to 0, while the voxels outside the lipofuscin mask remained unchanged. The background of the masked vGluT1 channel was subtracted. The surface bounding of vGluT1 boutons was smoothed at 0.15 μm and the diameter of the largest sphere fitting into the objects was taken at 0.5 μm. The masked vGluT2 channel was similarly surface-rendered, except that we fitted a Gaussian filter (SD = 0.3 μm) beforehand and used a user-defined fixed threshold of 68/255. The threshold for masked vGluT1 channel was determined by the Ridler and Calvard (R–C) algorithm and the resulting mean threshold was 20.1/255 for D98 and 26.2/255 for H01. We used Gaussian filter and user-defined threshold for the masked vGluT2 channel to effectively exclude the background and DCN neuron somata (Mao et al. 2018). The surface-reconstructed boutons were further filtered according to size by excluding surfaces with less than ten voxels (Haass-Koffler et al. 2012).

### Shrinkage and density calculation

The number of surface reconstructed boutons for each probe was exported and normalized to the corrected volume of probe in question. First, we obtained the (uncorrected) section thickness by adding the average diameter of the contours, 1.12 μm for vGluT1 and 1.19 μm for vGluT2 to account for boutons being only partly contained within a section (Braitenberg and Schüz 1998). The uncorrected probe volume was then obtained by multiplying the probe length (71.4 μm) and the probe width (71.4 μm) and the corrected depth (varying from probe to probe). Tissue shrinkage affected the thickness of the section and was, therefore, taken into consideration. To determine the extent of the shrinkage, four random positions were chosen within the DCN region for each stain section. All the values were then averaged and the cerebellar sections from D98 were shrunk to 24.6 μm thickness, while those of H01 were shrunk to a thickness of 26 μm, the original sectioning thicknesses being 40 μm for D98 and 50 μm for H01. Finally, we divided the uncorrected probe volume with 24.6/40 for D98 and 26/50 for H01 to obtain the corrected probe volumes.

### Calculating the co-labelling of vGluT1 and vGluT2 boutons

The quantification of co-labelling of vGluT1 and 2 was performed by obtaining the intersection of the surface boundaries of the 2 vGluT channels (vGluT1∩2). However, we could only perform channel subtractions in Imaris. We, therefore, subtracted vGluT1 from the vGluT1-only channel. The vGluT1-only channel was obtained for each probe by subtracting the vGluT2 channel from the original vGluT1 channel for the rats and from the lipofuscin cleaned vGluT1 channel in the case of the monkey. The number of the co-labelled boutons was divided by the vGluT1 bouton number or the vGluT2 bouton number to ascertain the co-labelled percentage for vGluT1 boutons or vGluT2 boutons. The percentages were compared between different nuclei and between the two species.

### Statistical analysis

The vGluT1 and 2 density and volume values were checked for normal distribution and variance homogeneity. Quantile–quantile plots (qqnorm in R, http://www.r-project.org) and Shapiro-tests (package lme4 in R) indicated normal distribution for vGluT1 and vGluT2 volume. Levene’s tests (package Rcmdr in R) indicated equal variance for vGluT1 and vGluT2 volume. The vGluT1 and vGluT2 density values were power transformed by transformTukey (package rcompanion in R) to secure normal distribution and equal variance. Since multiple data points were obtained in each subject (Aarts et al. 2014), a linear mixed effect model was used to accommodate the dependence of the data. The linear mixed effects model via restricted maximum likelihood estimations were performed using lme4 package in R. The response was the transformed density or non-transformed volume (for both vGluT1 and 2). The nuclei variance was modelled as a fixed effect, while subject variance was modelled as a random effect in the model. We also constructed a null model with only the random effect. We then compared the two models using a likelihood ratio test to ascertain whether these two models differed significantly from each other. Post hoc multiple comparisons (Tukey contrast) with Holm–Bonferroni correction were performed. The significance level α was set to 0.05. We also plotted the data with intensity color-coded scatter plots using matlab scripts (Eilers and Goeman 2004).

## Results

### vGluT1+ and vGluT2+ staining patterns in the cerebellum

**Fig. 1:**
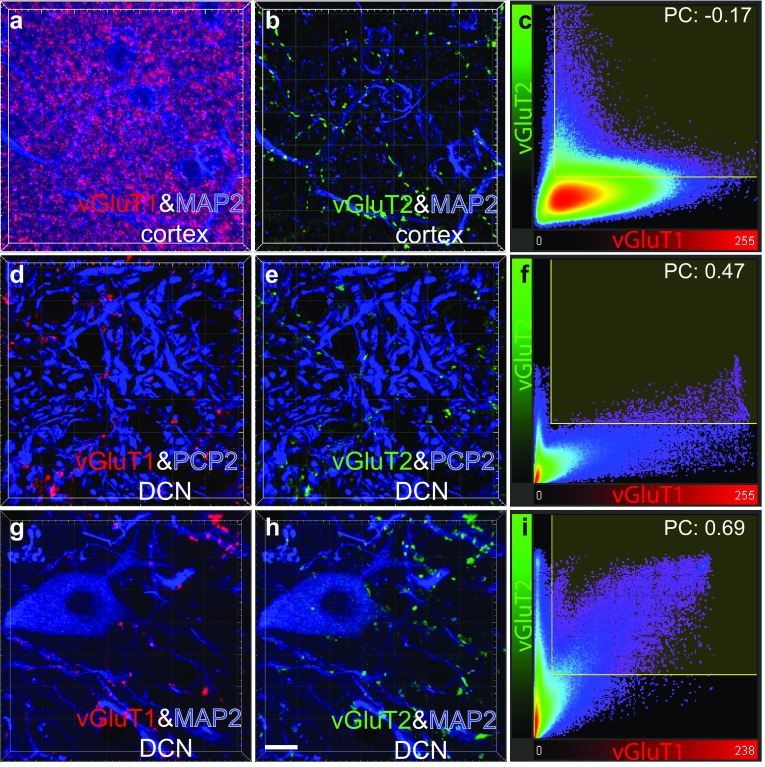
vGluT1+ and vGluT2+ staining patterns in the cerebellum. The staining pattern of vGluT1, vGluT2 and MAP2 in the cerebellar molecular layer is shown in **a**, **b**. The parallel fibers are detected by vGluT1 antibody (red), while the climbing fiber terminals are detected by vGluT2 antibody (green). The dendrites of Purkinje cells are labelled by MAP2 (Microtubule-associated protein 2, blue) (**a**, **b**). The colocalization analysis of vGluT1 and vGluT2 is shown in **c**, with Pearson’s coefficient (PC) − 0.17. The vGluT1, vGluT2 and PCP2 staining pattern in DCN is shown in **d**, **e**. The Purkinje cell axons are labelled by PCP2 in blue. The excitatory presynapses are labelled by vGluT1 (red) or vGluT2 (green) or both. The colocalization analysis of the vGluT1 and vGluT2 in DCN is shown in **f** and yields a PC of 0.47. Another example from the DCN is shown in **g**, **h**. The vGluT1, vGluT2 and MAP2 staining shows that the majority of excitatory boutons labelled by vGluT1 (red) or vGluT2 (green) contact the DCN dendrites labelled by MAP2 (blue) (**g**, **h**). The pearson’s coefficient of 0.69 indicates colocalization of vGluT1 and vGluT2 in the DCN (**i**). Scale bar in **h** is 10 μm and applies also to images (**a**, **b**, **d**, **e**, **g**)

The vGluT2 immunofluorescence showed a puncta pattern organized in a linear beaded fashion in the molecular layer (ML) and glomeruli-like pattern in the granule cells layer (GCL) of the cerebellar cortex (small arrows in Fig. S1a). There are also granular structures in the Purkinje cells layer (PCL) as described for lipofuscin in the literature [big arrow in Fig. S1a, (Brizzee et al. 1974)]. Not surprisingly, these structures are obtained again under our setting which is used for lipofuscin fluorescence (big arrow, Fig. S1b). The staining pattern of vGluT2 after lipofuscin channel subtraction is consistent with our previous study in the rat using the same antibody (Fig. S1c) (Mao et al. 2018). Preabsorption of vGluT2 antibody with its corresponding peptide abolished the vGluT2 signals in the cortex, pointing to the specificity of the vGluT2 antibody (Fig. S1d, e, f). Similarly, the vGluT2 antibody is also specific in the DCN region (Fig. S1 g–l). We also controlled the multiple staining patterns in the cerebellar cortex (molecular layer) and DCN of the monkey. As anticipated, parallel fibers were stained by vGluT1 only, whereas climbing fiber presynapses were stained by vGluT2 only (Fig. 1a, b). The vGluT1 and vGluT2 were separated from each other in the molecular layer, as the negative Pearson’s coefficient indicates (Fig. 1c). We also compared the staining of vGluT1 and 2 with the PCP2 staining in the DCN and found them to be segregated as in the molecular layer (Fig. 1d, e). However, unlike in the cortex, the vGluT1 and vGluT2 show some colocalization (Pearson’s coefficient 0.47). These co-labelled boutons may come from mossy fibers that express both vGluT1 and vGluT2 (Boulland et al. 2004). A comparison of labelling intensity within a voxel confirmed the different patterns; in contrast to the DCN there was no above-threshold intensity co-labelling of vGluT1 and 2 in the molecular layer (Fig. 1c, f). The spatial relationship of the vGluT1 and 2 labelled puncta with their postsynaptic partner, the dendrites from DCN neurons, was also examined in a quadruple staining environment. Most of the vGluT1 and 2 labelled puncta can be observed around the dendrites (Fig. 1g, h). Again, the colocalization of vGluT1 and vGluT2 in the DCN can be seen in this example (Fig. 1i).

### Comparison of vGluT1+ and vGluT2+ boutons within DCN

To obtain the quantitative data of vGluT1+ and vGluT2+ bouton density and compare between nuclei, the lipofuscin signal was first detected and eliminated from the vGluT1 and vGluT2 channels. First, we verified the lipofuscin excitation and emission characteristics in unstained macaque cerebellar sections (see supplementary material and Fig. S2). Then we validated our lipofuscin masking approach in the vGluT1 and vGluT2 channels and this is summarized in the supplementary material (see also Fig S2–4).

**Fig. 2:**
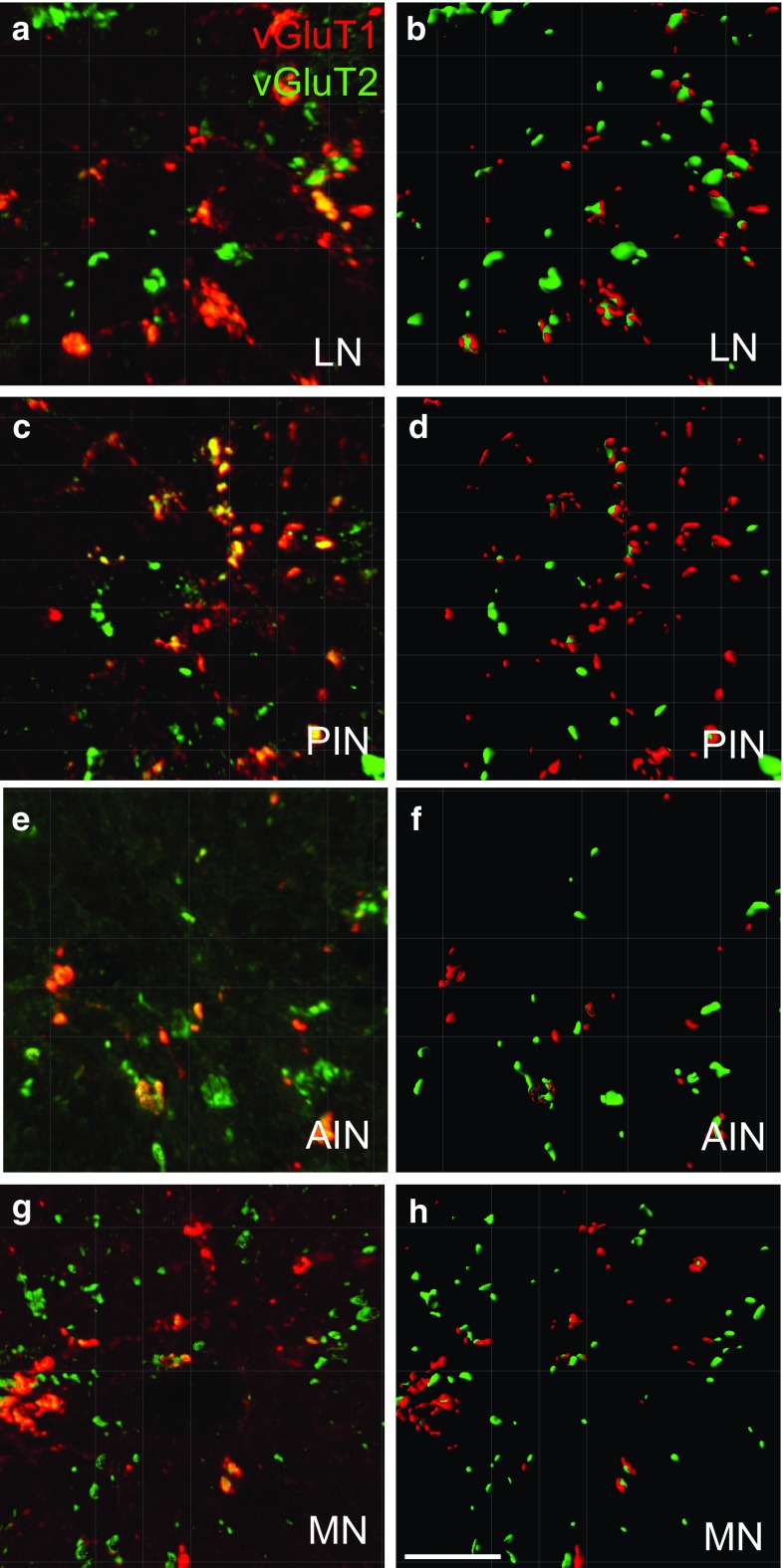
Examples of vGluT1 and 2 staining in the monkey DCN. Comparison of original microscopic views (**a**, **c**, **e**, **g**) and surface-rendered views (**b**, **d**, **f**, **h**) from vGluT1 and vGluT2 stained boutons in different nuclei. The Ridler–Calvard algorithm was used for the red channel, vGluT1, while the thresholding for vGluT2 was user defined at 68/255. Scale bar 10 μm in **h** applies to all other images as well

The structures that are labelled by vGluT1, vGluT2 or both are shown in different nuclei (Fig. 2). Double-labelled structures can be found in all the different nuclei. However, the vGluT1+ boutons are sparser in MN and AIN than in LN and PIN. Nevertheless, the vGluT2+ boutons are more similar between nuclei (Fig. 2a, c, e, g). The surface analysis allowed for a detailed visualization of vGluT1 and vGluT2-labelled structures and quantification of the number and volume of the boutons as well as the co-labelled boutons (Fig. 2b, d, f, h).

### vGluT1+ and vGluT2+ bouton density and volume within DCN

**Fig. 3:**
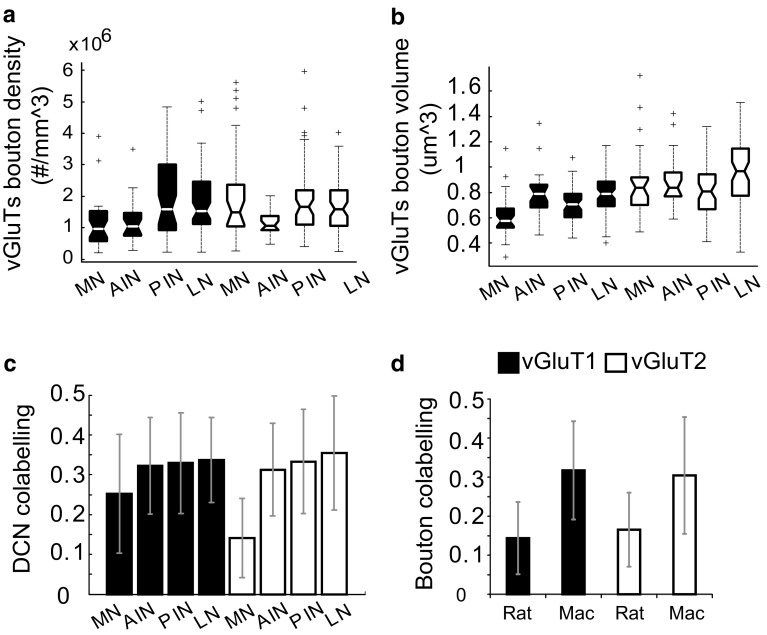
Boxplots of the density, volume and co-labelling of vGluT1- and vGluT2-stained boutons in different nuclei. **a**–**c** The results from the rhesus monkey DCN. **a** The density of vGluT1 and vGluT2 profiles. The vGluT1 density is highest in LN/dentate and PIN and lowest in MN and AIN (*p* = 1.87 × 10^−6^). The vGluT2 density is comparable between nuclei (*p* = 0.049) except for a lower density in the AIN. **b** The volumes of vGluT1 and 2 profiles. The mean vGluT1 and vGluT2 bouton volumes are 0.7 μm^3^ and 0.9 μm^3^, respectively. **c** Co-labelled percentage of vGluT1 and vGluT2 is compared in different nuclei of the monkey, with the average value of 30% for vGluT1 and 31% for vGluT2. **d** The co-labelling of vGluT1 and vGluT2 is significantly lower in the rat (around 15%) than in the monkey

We quantified the vGluTs bouton density in the different DCN. The vGluT1+ bouton density is higher in the PIN and LN, with 1.97 × 10^6^ (SEM = 1.78 × 10^5^) and 1.84^6^/mm^3^ (SEM = 4.96 × 10^5^), and lower in AIN and MN, 1.21 × 10^6^ (SEM = 3.04 × 10^5^) and 1.12 × 10^6^/mm^3^ (SEM = 3.3 × 10^5^) (ANOVA of mixed effect model, *df*: 3, Chi-square: 29.38, *p* 1.87 × 10^−6^). The vGluT2+ bouton density differed less intensely than the vGluT1 between subnuclei, although there was still a significant difference (ANOVA of mixed effect model, *df*: 3, Chi-square: 7.85, *p* 0.049), mainly due to the lower density of vGluT2 in AIN. The mean density for vGluT2 was 1.7^6^/mm^3^ (SEM = 8.29 × 10^4^) for the total DCN. The vGluT1 and 2+ bouton density data were power transformed to secure normal distribution and equal variance to satisfy the criterion for the statistic tests (Fig. S5a, c). The density and the volume of vGluT1+ and vGluT2+ boutons are shown for the four nuclei (Fig. 3a, b). The co-labelling of vGluT1 and vGluT2 is comparable in the AIN, PIN and LN, around 30%, with slightly lower values in MN (Fig. 3c). However, both the rat vGluT1 and vGluT2 co-labelling, which were obtained from our previous study, are significantly lower in the rat DCN than in the macaque DCN (Fig. 3d). The mean vGluT1+ and 2+ bouton volume is around 0.7 μm^3^ and 0.9 μm^3^, respectively (Fig. 3b). The vGluT1 volume was slightly larger in the AIN (0.78 μm^3^) and LN (0.8 μm^3^) than in the other DCN (MN: 0.61 and PIN: 0.70 μm^3^) and the vGluT2 volumes was larger in the LN (0.97 μm^3^) than in the other DCN (PIN: 0.84, AIN: 0.89, MN: 0.86 μm^3^), albeit with a larger variability. A one-way ANOVA was used to test the variance by different nuclei classification and the results are summarized in Table 2.

**Table 2:** Post hoc analysis (Tukey) of power transformed vGluT1+ and vGluT2+ bouton density and volume size

Threshold level	Dependent variables	*df*	Lambda	ICC	Chisq	*p* value	Post hoc test
vGluT1							
R-C (23/255)	Density	3	0.25	0.0526	29.38	1.87 × 10^−6^	MN and AIN < PIN and LN
R-C (23/255)	Volume	3	1	0.0338	35.69	8.69 × 10^−8^	MN < AIN; MN < PIN < LN
vGluT2							
68/255	Density	3	0.05	0.0526	7.85	0.049	n.s.
68/255	Volume	3	0.25	0.0284	9.47	0.023	PIN < LN

The *df* denotes degree of freedom; lambda denotes the power used for power transformation to ensure normality and equal variance and ICC is the intracluster correlation (indicates the amount of dependence in the data). *p* values were obtained by ANOVA. Post hoc test shows the comparisons which are statistically significant. *p* values were adjusted by Holm–Bonferroni correction

**Fig. 4:**
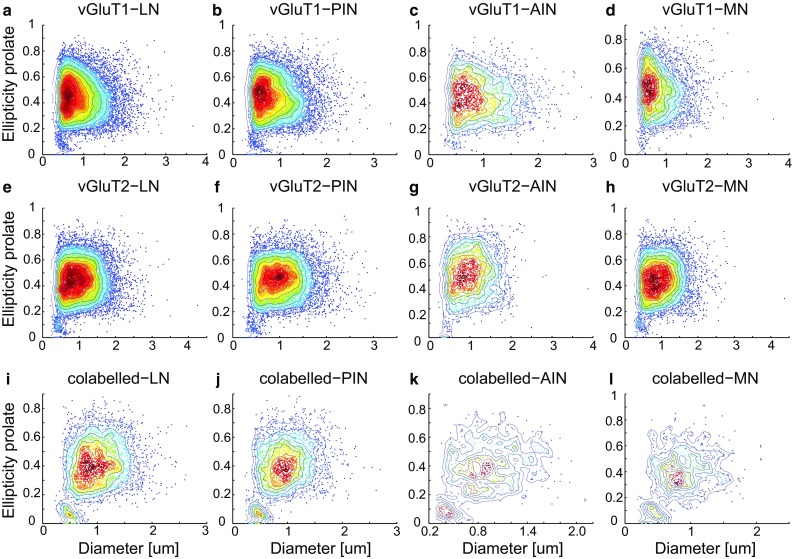
Intensity color-coded scatter plots. Scatter plots of two shape parameters (ellipticity prolate and diameter) and their relation to the profile diameters for vGluT1 (**a**–**d**) and vGluT2 (**e**–**h**). Plotted are the values for the different DCN subnuclei. The overall pattern is very similar, with one large cluster encompassing most of the profiles and one smaller cluster (to the lower left of the larger cluster) showing special subpopulations. The co-labelled boutons show similar shapes between nuclei (**i**–**l**). The color coding indicates the intensity of the scatter plot. Red represents higher and blue represents lower intensities

We also scrutinized our reconstructed vGluT1 and 2 boutons for any cluster outliers, such as axonal regions where the vGluTs are detected on their way to the presynaptic terminals (Mao et al. 2018). These elongated fragments would have a large ellipticity prolate value (close to 1) and a small diameter. Figure 4 displays density color-coded scatter plots of vGluT1 and 2 presynapse diameter vs. the shape parameter ellipticity prolate for the different DCN. Data show one large cluster with diameters larger than 0.5 μm and ellipticity prolate larger than 0.2 and one small cluster with ellipticity prolate smaller than 0.2. Based on these values they cannot be considered as axon fragments, because of their round shape. The number of boutons in the small cluster in the MN, AIN, PIN and LN amounted to 2.9%, 3.8%, 4.1%, 3.8% for vGluT1 and 4.2%, 3.8%, 3.6%, 4.2% for vGluT2, respectively. These boutons may come from a distinct population, e.g., the intrinsic DCN glutamatergic synapses.

### Number of vGluT1+ and 2+ bouton per neuron

**Fig. 5:**
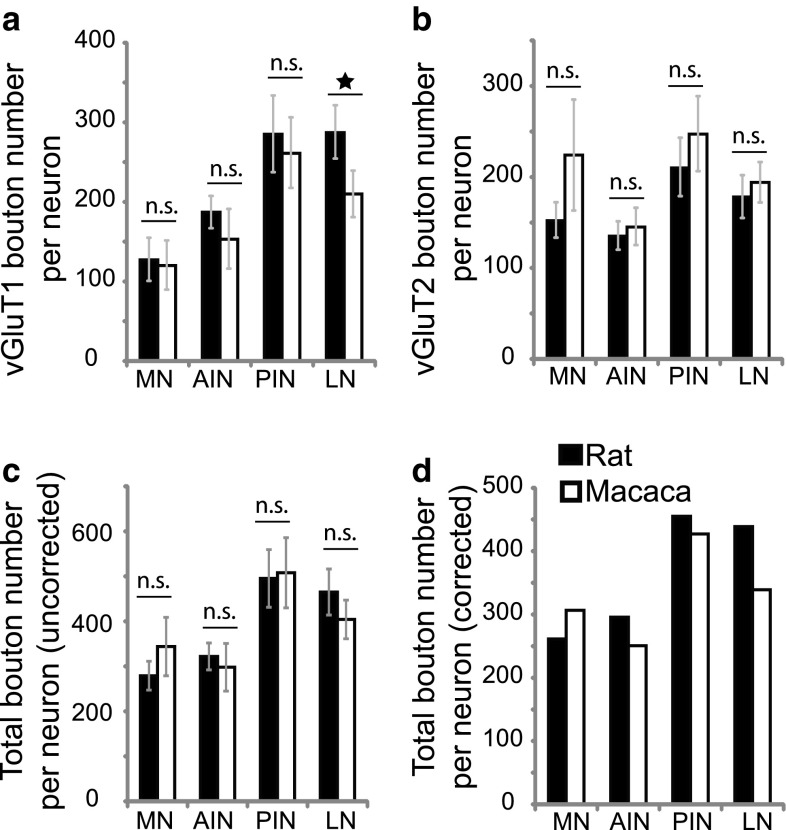
Bouton number per individual DCN neuron. The vGluT1- and vGluT2-labelled boutons are normalized by the neuronal density and compared in the rat and monkey DCN. The vGluT1 bouton number per neuron is similar in the rat and monkey, except in the LN in which the (95% confidence intervals) error bars do not overlap (**a**). The vGluT2-labelled bouton per neuron is similar in the rat and monkey DCN (**b**). The sum of vGluT1 and vGluT2 (uncorrected total) bouton number per neuron (**c**) was adjusted to remove the twice counted double-labelled boutons. This was done by subtracting the co-labelled vGluT1 bouton number per neuron from the total vGluT1 bouton number per neuron and then adding the total vGluT2 bouton number per neuron (**d**, “corrected”). In the monkey, the total bouton number per neuron is highest in the PIN, with a value of 427 boutons per neuron

We calculated the mean bouton number per DCN neuron on the basis of our vGluT1 and vGluT2 labelled boutons in the current study and the neuron density in our previous study (Hamodeh et al. 2014, 2017). The vGluT1 and 2 bouton number per neuron is similar in rat and monkey. However, the vGluT1 bouton number per neuron is significantly lower in the macaque LN/dentate (Fig. 5a). The total excitatory synaptic number per neuron was calculated by subtracting the co-labelled vGluT1 bouton number per neuron from the total vGluT1 bouton number per neuron and adding the total vGluT2 bouton number per neuron (Fig. 5c, d). The total bouton number per neuron in macaque DCN is lower than in the rat, except for the MN. Within the macaque, PIN has the largest bouton number per neuron (Fig. 5c, d).

## Discussion

In this study, we quantified vGluT1 and 2 in a region of a primate brain for the first time by also taking the confounding contribution of lipofuscin autofluorescence into account. Our quantification shows that the largest differences are observed between the DCN in the vGluT1 densities. This result is in agreement with our previous observation finding similar differences in the rat vGluT1 densities (Mao et al. 2018). This result also confirms that our observation of variation in the dendritic length density in the macaque DCN (Hamodeh et al. 2014, 2017) is accompanied by similar vGluT1 variations, i.e., regions with higher dendritic length densities show higher vGluT1+ densities. In addition, our results also point to some important differences between the rat and monkey LN/dentate, also confirming our previous dendritic quantifications showing hyposcalled dendrites in the LN/dentate. We will first discuss the potential biases and limitations of our method and then return to the interpretation of our results.

Our quantitative approach used systematic random sampling of series of multiple immunofluorescence-stained sections. In total we acquired 168 probes with 3D-stacks covering the monkey DCN from two monkeys. We adopted a new approach that involves recording an additional lipofuscin channel rather than using chemicals to diminish the lipofuscin autofluorescence. This channel was used to mask the lipofuscin fluorescence from the other fluorescence channels. Although the 405 nm wavelength laser used to excite the lipofuscins had the potential to bleach other fluorophores, we did not observe any indications that this was the case at our laser excitation powers. We generated surfaces from the lipofuscin fluorescence and used these to mask the other channels. When the lipofuscin regions are masked, their intensities change to zero. Therefore, if the structure of interest is also located in the cytoplasm, they could be affected. In our case, this did not occur since the glutamate transporters we quantified were located in the presynapses.

One of our main finding is that the density of vGluT1-labelled boutons is higher in phylogenetically newer parts of DCN, the LN/dentate and PIN, thus confirming our previous findings in the rat (Mao et al. 2018). This is also in agreement with our previous result of higher dendritic length density in these parts of the DCN. Nevertheless, the comparison of the vGluT1 densities showed that the monkey DCN (1.5 × 10^6^ vs. 6.1 × 10^6^) had a lower overall synaptic density of about 25% of the rat values (Mao et al. 2018). This is comparable to the reduction in neuronal density (8640 vs. 25,300) to 34% of the rat densities and shows that the overall vGluT1 synapses per neuron remain comparable. A comparison of the dendritic length density (Hamodeh et al. 2017) of monkeys vs. rats (51.5 vs. 39.8 m/mm^3^), shows a lower reduction to 77% of the rat values and would point to a general reduction in the number of vGluT synapses per dendritic length. Excitatory synapses to the DCN neurons largely originate from the precerebellar nuclei. The largest of these nuclei is the basilar pontine nuclei which increase considerably in size and neuron number in primates. Therefore, we would have anticipated a higher number of vGluT synapses per neuron in the LN/dentate and PIN.

### Comparable number of excitatory synapses per neuron in rat and monkey DCN

One explanation of the comparable values of excitatory synapses per neuron could be due to the modular organization of the cerebellum with similar number of units/neurons per module. However, other, potentially confounding factors may have led to a reduction of vGluT immunostaining in the monkey. The antibodies we used were optimized to stain the rodent’s vGluT c-terminal (unspecified peptide length for vGluT1 and amino acids 510–582 for vGluT2). A comparison of the amino acid sequence of the two transporters between rat and macaca mulatta shows that vGluT1 has an essentially conserved sequence, whereas the vGluT2 shows multiple amino acid changes in the last 72 amino acids. It is, therefore, possible that the vGluT2 immunostaining in the monkey tissue could be altered, which would also explain the higher threshold used for vGluT2 compared to the rat. However, a comparison of the staining pattern within the molecular layer did not show colocalization at above-threshold intensities for the vGluT2+ only climbing fibers and the vGluT1+ only parallel fibers. Our calculation of the individual excitatory inputs per neuron was based on different individuals and this may have introduced additional variability in our results. We used systematic unbiased sampling in both cases and tried to minimize the influence of such bias. Finally, our two monkeys, aged 13 and 18 years, may have already shown signs of age-related synapse elimination. As anticipated, our quantification of the lipofuscin did show a higher lipofuscin density in the older monkey (Gilissen et al. 2001). However, we did not observe systematically lower vGluT densities in the older monkey (D98 vs. H01:1.5 × 10^6^ vs. 2.3 × 10^6^/mm^3^ for vGluT1 and 1.8 × 10^6^ vs. 1.2 × 10^6^/mm^3^ for vGluT2). In summary, our data could point to the importance of a rather constant number of excitatory synapses per DCN neuron, reflecting a limited number of input units per DCN neuron.

### Increased vGluT1 and 2 co-labelling in the monkey DCN

One unexpected finding of our study is that the monkey DCN contain a higher percentage of vGluT1 and 2 co-labelled presynapses than those of the rat DCN (30 vs. 15%). This is probably due to the fact that the density of vGluT2 in the monkey is higher than in the rat. The cerebellum is one of the few brain regions that shows the presence of both kinds of vGluTs (in climbing and mossy fibers), as well as presynapses with both kinds of vGluTs in a subset of mossy fibers. Developmental studies have shown that, during the early stages of development, vGluT2 synapses change to vGluT1 following the switch of vGluT2 mRNA expression to vGluT1 mRNA expression in the neuron somata (Miyazaki et al. 2003). This suggests that a change occurs at the transcription and translation level of protein synthesis. This could thus imply that the mossy fibers retain a status comparable to that of the early stages of development, allowing for prolonged modification from vGluT2 to vGluT1. A different scenario emerges if we look at a recent learning/plasticity study in the DCN. The study by Boele and colleagues showed an increase in basilar pontine nuclei vGluT2 mossy fiber presynapses in the DCN following Pavlovian eyeblink conditioning (Boele et al. 2013). So far, it remains unclear whether this kind of learning in the DCN requires the sprouting and formation of new vGluT2 synapses, or whether it is achieved by an increase in vGluT2 levels in vGluT1 mossy fiber collaterals. The latter has not been described so far and, therefore, currently appears to be the less likely scenario.

### Hyposcaling of vGluT1+ inputs to the primates LN/dentate

A further new finding of our study is that there is a lower number of vGluT1+ excitatory synapses per neuron in the LN/dentate of the rhesus monkey compared to the rat’s LN/dentate. This result tallies well with the lower dendritic length per neuron that we already reported in the monkey LN/dentate (Hamodeh et al. 2017) and lends further support to our model of hyposcalled dendrites that underlie the special architecture of the LN/dentate. The hyposcaled dendritic arbors (Hamodeh et al. 2017) and vGluT1+ inputs (this study) could allow for an increase in the number of independent/segregated parasagittal strips in the cerebellar hemispheres connected to the LN/dentate, which would confirm a recent prediction (Jorntell 2017). A theory proposed by van Essen (1997) on how and why structures in the central nervous system tend to fold was based on the amount of mechanical tension that arises from the connectivity pattern of a given brain region (Van Essen 1997). If we assume that the hyposcalled dendrites with smaller region of influence and with less excitatory synaptic connections would lead to less tightly connected tissue, then these less tightly connected regions would lead to a reduction of tension, which in turn would lead to some indentation and folding at these sites during the ontogenetic and phylogenetic volume increase of the nucleus. These changes could then first lead to the emergence of a torus-like or cup-shaped nucleus, as indeed the rhesus monkey LN/dentate exhibits (Sultan et al. 2010), whereas, in the nucleus with more similar connectivity tension would be basically the same all across the nucleus and would help in preserving a globose structure.

## Significant statement

We have quantified the excitatory synapses by studying the vesicular glutamate transporter (vGluT) 1 and 2 in the primate cerebellar nuclei using a novel approach to mask lipofuscin autofluorescence. Our findings confirm our previous data from the rat of a higher vGluT1+ bouton density in the phylogenetically newer cerebellar nuclei of the macaque. We also find a similar number of excitatory boutons per neuron comparing rat and monkey pointing to a conserved modular organization of the cerebellum with similar number of neurons per module. Our findings also confirm our model of hyposcalled dendrites and vGluT1+ inputs to the specifically enlarged LN/dentate of primates.

## Electronic supplementary material

Below is the link to the electronic supplementary material.

Figure legend S1: Preabsorption test for vGluT2. In the cerebellar cortex the original vGluT2 channel containing lipofuscin not only detects the beaded-like climbing fiber terminals in the molecular layer (small arrow), the glomeruli structures in the granule layer (small arrow) but also the lipofuscin signals from Purkinje cells layer (big arrow) (a). The autofluorescence in the cerebellar cortex is obtained in a separate channel using excitation at 405 nm and emission filters at 650-750 nm (b). After channel subtraction, the structures detected by vGluT2 antibody is confined to the molecular and granule layer (c). In contrast, after applying the vGluT2 antibody and the peptide antigen mixture we only detect the autofluorescence in the Purkinje cell layer (d and e). No signal was left after subtracting lipofuscin channel from the vGluT2 channel (f). In the DCN, bouton-like structures and big granules were detected in the vGluT2 channel (g). The autofluorescence was also present in DCN (h). Only puncta structures were left after channel subtraction (i). The preabsorption test also abolished the vGluT2 staining in the DCN (j-l). Scale bar in (f): 20 μm and applies also to (a-e). Scale bar in (l): 10um and applies to (g-k). Results of masking lipofuscin in vGluT1 and vGluT2 channels. We scanned unstained macaque cerebellar sections by exciting the tissue at 405 nm and by collecting the emission from 650-750 nm and detected fluorescent granule-like structures (Fig. S2a). When the specimens were scanned in lambda mode, these granule-like structures showed a broad emission spectral range from 450 to 750 nm and peaking at around 530 nm. This indicates that these structures are lipofuscin (Fig. S2b). Since the lipofuscin has not only a broad emission spectrum but also broad excitation spectra, granule-like structures were also obtained under the excitation of 633 nm laser (Fig. S2c). Again these structures have broad emission spectra under lambda mode (Fig. S2d). However, independent of the granules’ location (inside the neuronal soma ROI 3, Fig. S2a or outside the soma ROI 1,2 and 4, Fig. S2a), they share the same fluorescence emission properties, thus further endorsing the compositional homogeneity of these structures. By contrast, the intensity of tissue background fluorescence was lower than the lipofuscin fluorescence and dropped to baseline at longer wavelengths (ROI 5, Fig. S2a and b, ROI 2, Fig. S2c and d). The lipofuscin fluorescence can also be well segregated from the tissue background fluorescence from the emission range of 650-750 nm (light green rectangle in Fig. S2b and d). We then tested whether the lipofuscin fluorescence can be segregated from our staining. Alexa 405 was used to detect Purkinje cell axons in our quadruple staining with optimal excitation at 405 nm. The emission spectrum of the Alexa 405 and lipofuscin differed considerably. The emission range was 405-600 nm with the peak around 450 nm for Alexa 405. The Alexa 405 emission range was much narrower than that of lipofuscin, and most importantly, there was no emission from 650-750 nm, indicating that exciting the lipofuscin at 405 nm and obtaining its emission within the range 650-750 nm would result in a pure signal (Fig. S2e-f). This configuration ensured no bleed-through of fluorescence emission of the rest of the fluorochromes into the lipofuscin emission signal. In a next step, we needed to remove the lipofuscin staining from the vGluT1 and vGluT2 signal. We used the lipofuscin channel to produce a mask (Fig. S3a), by creating surfaces fitted to the lipofuscin granules (Fig. S3b). The quantification of lipofuscin volume in DCN from two monkeys was also obtained on the basis of the constructed surfaces. The amount of lipofuscin in the DCN of the 18-year-old D98 is about two-fold that of the 13-year-old H01. However, the amount of lipofuscin in the subnuclei does not differ (Fig. S4). The lipofuscin mask was subtracted from the vGluT1 and vGluT2 channels and we obtained the masked vGluT1 and vGluT2 channels, respectively (Fig. S3c, e). Finally, the lipofuscin-removed vGluT1 and vGluT2 channels were surface-rendered for quantification (Fig. S3d, f) (EPS 35179 kb)

Figure legend S2: Lipofuscin fluorescence detection and its spectral characteristics. The lipofuscin fluorescence in the unstained DCN was present both in the neuronal cytoplasm (ROI 3) and outside the neuron (ROI 1, 2, and 4) under excitation of 405 nm (a). The emission spectrum of each region of interest (ROI) selected is shown in (b). The ROI 5 was tissue background. The spectra of ROI 1-4 were widely spread from 411 nm to 752 nm, albeit with varying intensities. The maximum emission intensity under 405 nm excitation was around 530 nm (b). The unstained section can also be excited at 633 nm (c) and the spectra are shown in (d). The quadruple stained rhesus monkey section was scanned under 405 nm excitation in lambda mode (e). The fluorescence spectra from PCP2/Alexa 405 and lipofuscin differ considerably: ROI of 4 and 5 are from PCP2 stained structures and ROI 1-3 and 7-8 are from lipofuscin (e, f). In the emission region of 650-750 nm, the PCP2/Alexa 405 was reduced to baseline, while lipofuscin still emits a high-intensity signal (f). The light green columns in (b) and (d) indicate the emission range 650-750 nm. The ROI 6 was tissue background. Scale bar in (a), (c) and (e) is 10um (EPS 23883 kb)

Figure legend S3: Workflow to remove lipofuscin autofluorescence (AF) from vGluT1 and vGluT2 channels. The lipofuscin channel signal (a) was surface-rendered in Imaris (b). This mask was then used to subtract from the original vGluT1 and vGluT2 channels (c, e) to obtain the masked vGluT1 and vGluT2 channel (d, f). Scale bar in (f) and in all other images is 10um (EPS 3923 kb)

Figure legend S4: Lipofuscin quantification in DCN. The volume of the lipofuscin surfaces was obtained and compared in different nuclei of two monkeys (D98 (a) and H01 (b)). The lipofuscin volume fraction was higher in the older monkey (D98 i.e., a). Lipofuscin density did not differ significantly between the DCN (ANOVA, p = 0.28 for D98, p = 0.27 for H01). Examples of microscopic images with lipofuscin (red) is shown in (c) and (d) with vGluT1 channel (green) (EPS 8090 kb)

Figure legend S5: Quantile–quantile plots of the power transformed data (a–d). The Shapiro *p* values in (a) (c)and (d) are larger than 0.05, indicating that the transformed vGluT1 and 2 density and vGluT2 volume are derived from normally distributed populations. The vGluT1+ bouton volume data was not power transformed (b) (EPS 875 kb)

Supplementary material 6 (DOCX 12 kb)
