# Supplementary material for: Quantitative organization of the excitatory synapses of the primate cerebellar nuclei: further evidence for a specialized architecture underlying the primate cerebellum
Source: Brain Struct Funct. 2019 May 17;224(6):1987–98. doi: 10.1007/s00429-019-01888-8 (PMC6591209; doi:10.1007/s00429-019-01888-8)
Supplement: Supplementary file 6 — Supplementary material 6 (DOCX 12 kb) [file 429_2019_1888_MOESM6_ESM.docx]

### Results of masking lipofuscin in vGluT1 and vGluT2 channels

We scanned unstained macaque cerebellar sections by exciting the tissue at 405nm and by collecting the emission from 650-750nm and detected fluorescent granule-like structures (Fig. S2a). When the specimens were scanned in lambda mode, these granule-like structures showed a broad emission spectral range from 450 to 750nm and peaking at around 530nm. This indicates that these structures are lipofuscin (Fig. S2b). Since the lipofuscin has not only a broad emission spectrum but also broad excitation spectra, granule-like structures were also obtained under the excitation of 633nm laser (Fig. S2c). Again these structures have broad emission spectra under lambda mode (Fig. S2d). However, independent of the granules’ location (inside the neuronal soma ROI 3, Fig. S2a or outside the soma ROI 1,2 and 4, Fig. S2a), they share the same fluorescence emission properties, thus further endorsing the compositional homogeneity of these structures. By contrast, the intensity of tissue background fluorescence was lower than the lipofuscin fluorescence and dropped to baseline at longer wavelengths (ROI 5, Fig. S2a and b, ROI 2, Fig. S2c and d). The lipofuscin fluorescence can also be well segregated from the tissue background fluorescence from the emission range of 650-750nm (light green rectangle in Fig. S2b and d). We then tested whether the lipofuscin fluorescence can be segregated from our staining. Alexa 405 was used to detect Purkinje cell axons in our quadruple staining with optimal excitation at 405nm. The emission spectrum of the Alexa 405 and lipofuscin differed considerably. The emission range was 405-600nm with the peak around 450nm for Alexa 405. The Alexa 405 emission range was much narrower than that of lipofuscin, and most importantly, there was no emission from 650-750nm, indicating that exciting the lipofuscin at 405nm and obtaining its emission within the range 650-750nm would result in a pure signal (Fig. S2e-f). This configuration ensured no bleed-through of fluorescence emission of the rest of the fluorochromes into the lipofuscin emission signal. In a next step, we needed to remove the lipofuscin staining from the vGluT1 and vGluT2 signal. We used the lipofuscin channel to produce a mask (Fig. S3a), by creating surfaces fitted to the lipofuscin granules (Fig. S3b). The quantification of lipofuscin volume in DCN from two monkeys was also obtained on the basis of the constructed surfaces. The amount of lipofuscin in the DCN of the 18-year-old D98 is about two-fold that of the 13-year-old H01. However, the amount of lipofuscin in the subnuclei does not differ (Fig. S4). The lipofuscin mask was subtracted from the vGluT1 and vGluT2 channels and we obtained the masked vGluT1 and vGluT2 channels, respectively (Fig. S3c, e). Finally, the lipofuscin-removed vGluT1 and vGluT2 channels were surface-rendered for quantification (Fig. S3d, f).
